# Lymph node dissection in atypical endometrial hyperplasia

**DOI:** 10.4274/jtgga.2017.0043

**Published:** 2017-09-01

**Authors:** Salih Taşkın, Özgür Kan, Ömer Dai, Elif A. Taşkın, Kazibe Koyuncu, Ayşegül Alkılıç, Mete Güngör, Fırat Ortaç

**Affiliations:** 1 Department of Obstetrics and Gynecology, Ankara University School of Medicine, Ankara, Turkey; 2 Department of Obstetrics and Gynecology, Losante Hospital, Ankara, Turkey; 3 Department of Obstetrics and Gynecology, Acıbadem University School of Medicine, İstanbul, Turkey

**Keywords:** Atypical hyperplasia, concomitant, endometrial carcinoma, lymphadenectomy

## Abstract

**Objective:**

The rate of concomitant endometrial carcinoma in patients with atypical endometrial hyperplasia is high. We aimed to investigate the role of lymphadenectomy in deciding adjuvant treatment in patients with concomitant atypical endometrial hyperplasia and endometrial carcinoma.

**Material and Methods:**

Women with atypical endometrial hyperplasia were enrolled in this retrospective study. Lymph node dissection was performed in only some patients who gave informed consent if their surgeon elected to do so, or if the intraoperative findings necessitated. The final histopathologic evaluations of surgical specimens were compared with endometrial biopsy results.

**Results:**

Eighty eligible patients were evaluated. Seventy-two (90%) patients had complex hyperplasia with atypia, and 8 (10%) patients had simple hyperplasia with atypia. Hysterectomy and bilateral salpingo-oophorectomy were performed to all patients; 37 also underwent lymph node dissection. Lymph node dissection was extended to the paraaortic region in 9 of 37 patients. The concomitant endometrial carcinoma rate was 50%. Two patients had lymph node metastasis. Among 40 cases of carcinoma, 17 had deep myometrial invasion and/or cervical or ovarian involvement or grade 2 tumors with superficial myometrial invasion on hysterectomy specimens; 27.5% of all carcinomas were stage Ib or higher.

**Conclusion:**

The concomitant endometrial carcinoma rate was high in patients with atypical endometrial hyperplasia. Nearly half of these patients had risk factors for extrauterine spread. Lymph node dissection might be helpful to decide adjuvant treatment.

## INTRODUCTION

The rate of concomitant endometrial cancer in patients with atypical endometrial hyperplasia (AEH) is high in hysterectomy specimens. Although some factors have been suggested to predict concomitant endometrial cancer such as older age, diabetes and obesity (1), there is no tool to predict concomitant malignancy precisely, and the vast majority of cases are diagnosed postoperatively in hysterectomy specimens. Moreover, intraoperative frozen section assessment with high accuracy is not available in most centers.

Postoperative histopathologic findings may be discordant to either pre- or intra-operative diagnoses. This issue makes the extent of surgery for AEH controversial, as it is in endometrial carcinoma. Hysterectomy may be insufficient in the event of concomitant carcinoma, especially if the patient has high risk factors, and lymph node status should be known to plan adjuvant treatment. Many reports underline the possibility of lymph node metastases, which cannot be ignored (2), and some surgeons recommend lymphadenectomy (3).

In the present study, we examined the rate of concomitant carcinoma, risk factors for extrauterine spread, and the role of lymph node dissection in deciding adjuvant treatment in patients with postoperative diagnosis of invasive carcinoma among patients who underwent surgery because of AEH, some including lymph node dissection.

## MATERIAL AND METHODS

The medical records from between January 2006 and December 2014 were reviewed and eighty patients who were diagnosed as having complex or simple AEH according to the World Health Organization classification system on endometrial biopsy were enrolled in the study. All endometrial biopsies were obtained with suction curettage (Pipelle) and all specimens were placed in formalin before the pathologic examination. Only patients who underwent hysterectomy without prior medical treatment for hyperplasia were eligible for this retrospective study. Patients with preoperative diagnoses of concomitant endometrial hyperplasia and endometrial carcinoma or who were treated with progestins were excluded. This study was designed as a retrospective data assessment; therefore, ethics committee approval was not required.

Electronic medical records, pathology reports, hospital and outpatient medical charts were reviewed. Data collected included age, preoperative diagnosis, medical treatment, type of operation, postoperative diagnosis, and final pathology.

Operations were performed by laparotomy (n=48) or laparoscopy (n=32) under general anesthesia. Lymph node dissection was performed if the surgeon elected to do so, or according to intraoperative findings such as an appearance suggestive of endometrial carcinoma during macroscopic observation of the opened uterus or suspicious metastatic lymph node(s). Frozen section evaluation of the uterus was not performed. Patients were informed about the risk of concomitant carcinoma and they gave informed consent for lymph node dissection if required.

The final histopathologic evaluation of surgical specimens and preoperative endometrial biopsy results were compared.

## RESULTS

Five hundred thirty-seven patients with endometrial hyperplasia were reviewed. One hundred twelve patients had AEH and 80 (71.4%) underwent surgery. The mean age of these 80 patients was 58.9 years. Seventy-two (90%) patients had complex hyperplasia with atypia, and 8 (10%) had simple hyperplasia with atypia. Forty-three (53.7%) patients underwent hysterectomy with bilateral salpingo-oophorectomy, and 37 (46.2%) had hysterectomy, bilateral salpingo-oophorectomy, and bilateral pelvic lymph node dissection; lymph node dissection was extended to the paraaortic region in 9 of these 37 patients.

The final pathology results of the surgical specimens and comparison of preoperative findings are shown in Table 1. Forty (50%) patients were diagnosed as having endometrioid-type endometrial carcinoma in the final pathologic examination of surgical specimens. The characteristics of 40 endometrial carcinoma cases according to the International Federation of Gynecology and Obstetrics 2009 Endometrial Carcinoma Staging System and lymph node metastases are given in Table 2. The histologic type in all patients with endometrial carcinoma was endometrioid. There were no grade 3 tumors.

Eight patients with simple AEH underwent surgical treatment. In the final histopathologic diagnosis, 4 patients had benign histology, 1 had simple hyperplasia without atypia, 2 had simple hyperplasia with atypia, and 1 of these 8 patients (12.5%) had stage Ia endometrioid-type endometrial carcinoma on hysterectomy specimens.

Fifteen (20.8%) of 72 patients with atypical complex endometrial hyperplasia had benign histology, 2 (2.7%) had simple hyperplasia without atypia, 4 (5.4%) had complex hyperplasia without atypia, and 39 (54.1%) had endometrioid carcinoma in their hysterectomy specimens.

**Figure 1:**
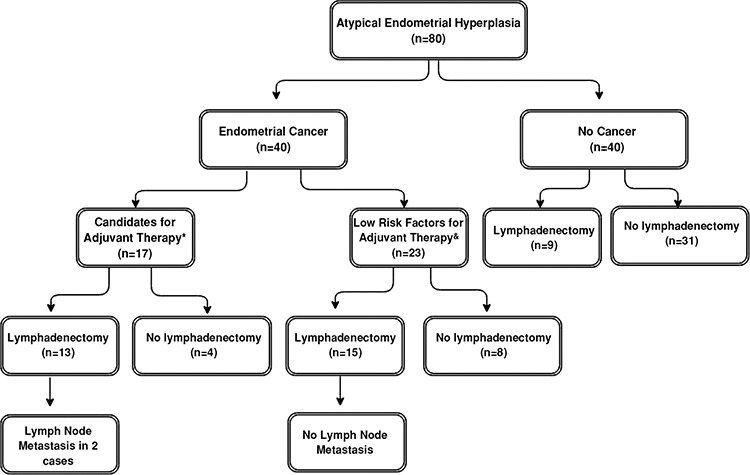
Brief summary of patient features *: Presence of at least one of the following on hysterectomy and salpingo-oophorectomy specimen: (i) Grade 2 with inner half myoinvasion, (ii) Deep myoinvasion (iii) Cervical stromal invasion (iv) Ovarian involvement *&: Grade 1 with inner half myoinvasion*

Among the 37 patients who underwent lymph node dissection, 28 were diagnosed as having endometrial carcinoma, and the remaining 9 had no invasive disease. As shown in Figure 1, the majority of patients were candidates for adjuvant treatment and underwent lymph node dissection. Four patients with grade 2 disease (3 with superficial myoinvasion and one with deep myoinvasion) received no lymph node dissection. These 4 patients were given adjuvant brachytherapy. Two of the 4 patients with stage Ib disease who underwent lymph node dissection were given brachytherapy; the other 2 were followed up without adjuvant treatment. Patients with stage II and IIIa disease were given pelvic radiotherapy. Two patients with stage IIIc disease had grade 2 tumors with deep myoinvasion and were given chemotherapy with extended radiotherapy.

## DISCUSSION

Some patients with AEH who are postoperatively diagnosed as having concomitant endometrial cancer carry high risk factors that necessitate knowledge about lymph node status in order to plan adjuvant therapies. Therefore, we evaluated the role of lymph node dissection in patients with AEH as a disease that carries a high risk for concomitant malignancy, which is preoperatively unpredictable in most patients.

In this study, we found that the prevalence of concomitant carcinoma in hysterectomy specimens was 50% in patients with AEH. This rate was 54.1% in patients with complex atypical hyperplasia and 12.5% with simple atypical hyperplasia. Among women with complex atypical hyperplasia diagnosed at endometrial biopsy, 17-52% were found to have concomitant endometrial carcinoma in previous studies (4, 5, 6, 7, 8, 9, 10). The concomitant carcinoma rate in our study is similar to these reports. The mean age of our patients was 58.9 years, which might explain the higher rate of concomitant endometrial carcinoma compared with other studies that were conducted on younger patient groups (8, 11, 12).

Invasive endometrial carcinoma concurrent with AEH is supposed to be grade 1 and associated with low risk factors, and hysterectomy was thought to be sufficient (13). However, some studies demonstrated that this was not the case. Whyte et al. (3) reported the rate of concurrent carcinoma as 29%, of which, 84% had myometrial invasion and 8% had cervical stromal invasion. In that series, one (5.5%) of 18 patients who underwent lymph node dissection had metastases. The authors concluded that the surgical staging decision, in addition to hysterectomy, was critical, especially in patients with complex atypical hyperplasia due to high rates of concomitant invasive cancer with high risk factors for extrauterine spread.

In a recent study, the ratio of lymph node metastases was estimated as 6.8% in patients with invasive endometrial carcinoma concomitant to AEH based on defined risk factors (2). This ratio decreases to 2.1% if all patients with AEH cases in the series are considered. Similarly, in our study, 2 of 28 (7.1%) patients with endometrial carcinoma who underwent lymph node dissection had lymph node metastasis. Therefore, the incidence of lymph node metastases in patients with concomitant endometrial carcinoma is nearly similar to clinical early-stage endometrial carcinoma.

Fifty percent of the women who were primarily diagnosed as having AEH had undiagnosed carcinoma. That kind of high coexistence rate may lead to inadequate surgical staging (8). In our study, all carcinomas had endometrioid histology and none of the tumors were poorly differentiated. All tumors were grade 1 or 2 (55% grade 1, 45% grade 2). In nearly 70% of carcinoma cases, tumor was confined to the inner half of the myometrium. On this account, lymphadenectomy for all AEH may not be advisable.

On the other hand, 17 patients with carcinoma had deep myometrial invasion, and/or cervical or ovarian involvement or grade 2 tumors with superficial myometrial invasion, and 27.5% of all carcinoma cases were stage Ib or higher. Thus, in 42.5% (17 of 40) of patients with endometrial carcinoma, and in 21% of all patients (17 of 80), lymph node dissection could have provided information about lymph node status in our study. Thirteen of these patients underwent lymph node dissection, and in the majority (except only 2), no lymph node metastasis was detected (Table 2). By these means, treatment plans were made more appropriately. Similarly, it was reported that lymph node dissection affected the adjuvant treatment decision in 28% of patients who were diagnosed as having endometrial carcinoma in uterine pathology without additional morbidity. The authors recommended lymph node dissection for AEH due to the high risk of concomitant carcinoma (3).

At this stage, routine lymphadenectomy in AEH cannot be recommended, it may be an unnecessarily aggressive approach. However, our results showed that extensive evaluation of patients with AEH is mandatory pre- and intraoperatively, according to the center’s capability. Immunohistochemical studies using phosphatase and tensin homolog (PTEN) and ARID1a on curettage materials can be helpful to exclude concomitant endometrial carcinoma. Loss of these markers may support the existence of concomitant carcinoma (14, 15).

Until now, there is no method to precisely determine lymph node status in endometrial carcinoma, other than lymph node dissection. We routinely perform pelvic lymph node dissection in all patients with endometrial carcinoma because lymph node dissection is the only accurate way to detect lymph node metastasis. Half of patients with endometrial carcinoma with pelvic lymph node metastasis also have paraaortic lymph node involvement (16). Paraaortic lymph node dissection is added when preoperative high risk factors or intraoperative palpable/suspicious pelvic and/or paraaortic lymph nodes are present. In the current study, pelvic nodes looked suspicious macroscopically, and dissections were extended to paraaortic area in two cases with lymph node metastasis (stage IIIc2).

Discordance between pre-and post-operative pathologic diagnoses can be encountered in up to 30% of patients (17). In a series of patients with preoperative diagnosis of grade 1 endometrium carcinoma, surgical staging affected the decision of adjuvant treatment in 29% of cases; in other words, the patients were protected against under or over treatment (18). Another study reported that 15% of preoperative grade 1 cases were upgraded, and 18% had high-risk uterine pathology (19). Surgical staging allows the identification of patients who woud benefit from adjuvant therapy, as well as those who may be safely spared from the morbidity of these treatments.

Nevertheless, lymph node dissection brings with it some additional surgical risks. Some groups tried to identify a subset of patients who could be spared from surgical staging. In some reports, frozen section examination was advised for determining the extent of the surgery; however, there have been conflicting reports about the accuracy of findings. Some authors stated that frozen section was useful for guiding intraoperative decision-making (20), whereas others found frozen section results as inconsistent with the final histopathologic examination results (21). Another study showed only 48% concordance between intraoperative frozen section and postoperative pathology in AEH, and the sensitivity for the diagnosis of endometrial carcinoma was reported as 33% (3). A model for predicting endometrial cancer based on age, body mass index, endometrial thickness, and postmenopausal status was proposed with 80% sensitivity and 70% specificity (22).

The Mayo Clinic suggests grouping patients with grade 1 and 2 tumors smaller than 2 cm diameter with myometrial invasion <50% as the low risk group and to skip lymphadenectomy (23). However, only specialized and experienced gynecologic pathologists can reliably define uterine risk factors in frozen sections. In practice, it is impossible for most oncology centers to employ such experts. Determining lymphadenectomy need is crucial because of these variable findings. Sentinel lymph node biopsy can be an option to evaluate lymph node status in patients with a preoperative AEH diagnosis. This method may overcome the problem related with frozen section accuracy and avoid re-operations in patients diagnosed as having high-risk endometrial cancer in the final pathology.

All our patients received their diagnoses after endometrial biopsies, rather than with dilatation and curettage, which can enable physician to sample larger portions of the endometrial cavity. This issue may underlie the high rate of concurrent carcinoma. The rates of concurrent carcinoma in patients diagnosed as having AEH via dilatation and curettage or via endometrial biopsy were reported as 17-30% and 43-45%, respectively (2, 3). Although one third of patients with concurrent carcinoma were still missed, dilatation and curettage before hysterectomy may be recommended for patients whose disease is diagnosed via endometrial biopsy.

The retrospective design and lack of comparison of perioperative outcomes according to lymph node dissection are the main limitations of our study. However, this study was focused on the role of lymph node dissection in deciding adjuvant treatment of endometrial carcinoma diagnosed in hysterectomy specimens of patients with AEH, rather than perioperative outcomes.

In conclusion, data to support lymph node dissection in all patients with AEH is lacking. Even in patients with endometrial carcinoma, the necessity for routine lymph node dissection is still under debate. However, the high rate of concomitant endometrial carcinoma with risk factors for extrauterine spread in a considerable number of patients with AEH should be kept in mind. Patients should be informed about this risk, and the value of lymph node dissection in patients with AEH should be demonstrated with large-scale studies in the near future.

## Figures and Tables

**Table 1:**
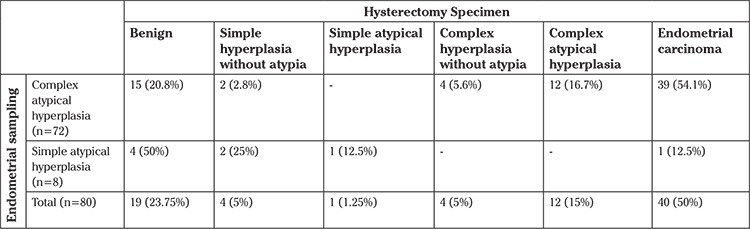
Comparison of pathological results of endometrial sampling and hysterectomy specimen of all cases (n=80)

**Table 2:**
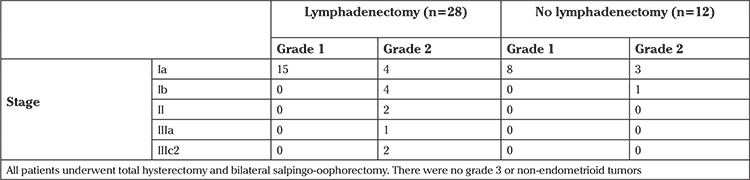
Endometrial cancer cases (n=40)
